# BIOFACQUIM: A Mexican Compound Database of Natural Products

**DOI:** 10.3390/biom9010031

**Published:** 2019-01-17

**Authors:** B. Angélica Pilón-Jiménez, Fernanda I. Saldívar-González, Bárbara I. Díaz-Eufracio, José L. Medina-Franco

**Affiliations:** Department of Pharmacy, National Autonomous University of Mexico, Mexico City 04510, Mexico; angiepilon96@gmail.com (B.A.P.-J.); felilang12@gmail.com (F.I.S.-G.); debi_1223@hotmail.com (B.I.D.-E.)

**Keywords:** chemical space, chemical data set, chemoinformatics, consensus diversity plot, drug discovery, molecular diversity, visualization

## Abstract

Compound databases of natural products have a major impact on drug discovery projects and other areas of research. The number of databases in the public domain with compounds with natural origins is increasing. Several countries, Brazil, France, Panama and, recently, Vietnam, have initiatives in place to construct and maintain compound databases that are representative of their diversity. In this proof-of-concept study, we discuss the first version of BIOFACQUIM, a novel compound database with natural products isolated and characterized in Mexico. We discuss its construction, curation, and a complete chemoinformatic characterization of the content and coverage in chemical space. The profile of physicochemical properties, scaffold content, and diversity, as well as structural diversity based on molecular fingerprints is reported. BIOFACQUIM is available for free.

## 1. Introduction

The significance of compound databases in drug discovery projects is continuously increasing. In fact, compound databases and chemical data sets are a centerpiece in pharmaceutical companies and other academic and government research centers [1]. In addition to their role in compound databases, natural products have been a major resource in drug discovery [2,3]. As reviewed elsewhere, there are several drugs recently approved for clinical use that are natural products or synthetic analogues of hit compounds initially identified from natural sources. A notable example is the fungi metabolite migalastat (Galafold®), approved in 2018 for the treatment of the Fabry disease [4]. Not unsurprisingly, natural product-based drug discovery is being coupled with other major drug discovery strategies such as high-throughput screening and virtual screening. Natural products are again gaining attention in the scientific community to address novel and/or difficult molecular endpoints, for instance, epigenetic targets [5,6].

Several compound databases of natural products have been constructed, curated and often maintained by academic and other not-for-profit research groups. Notable examples are the Universal Natural Product Database (UNPD) [7] and the Traditional Chinese Medicine (TCM) Database@Taiwan [8]. Of note, UNPD is no longer available online but represents the efforts of an academic group to assemble a large natural product database. Reference [4] confirms that there are other compound databases that collect natural products from specific geographical areas and countries, such as NuBBE_DB_ for natural products from Brazil [9] VIETHERB: A Database for Vietnamese Herbal Species was recently released to the public [10]. Other databases of natural products are discussed elsewhere [11,12,13]. Despite the fact that Mexico also has high levels of biodiversity, there are limited efforts to assemble a compound database of natural products. One example is UNIQUIM, recently reviewed by Medina-Franco [11].

The objective of this work is to introduce BIOFACQUIM as one of the first compound databases of natural products isolated and characterized in Mexico. In this proof-of-concept study, we discuss the assembly of the first version of this chemical data set along with a chemoinformatic characterization of molecular diversity, scaffold content and coverage in chemical space. The compound database is freely available via the web-interface BIOFACQUIM Explorer (https://biofacquim.herokuapp.com/), and is part of an initial effort towards building, updating and maintaining a compound database representative of the biodiversity of Mexico. Compounds in BIOFACQUIM are also available from ZINC15 at http://zinc15.docking.org/catalogs/biofacquimnp/

## 2. Materials and Methods

### 2.1. BIOFACQUIM Database

The database of natural products was assembled from a literature search. For the construction of the first version of BIOFACQUIM, the Scopus database (www.scopus.com) was searched using the keywords “natural products” and “School of Chemistry of the National Autonomous University of Mexico (FQ, UNAM)”. This search led to a list of scientific papers and researchers that work with natural products. The eight journals that had contributed the most thus far were selected: *Journal of Ethnopharmacology*, *Natural Products Research*, *Journal of Agricultural and Food Chemistry*, *Journal of Natural Products*, *Planta Medica*, *Phytochemistry*, *Natural Product Letters*, and *Molecules*. As part of the search, three filters were used for the selection of the articles in each journal. The first filter was the search by institution (FQ, UNAM), the second was the search by publication year (2000–2018), and the last was the detailed analysis of the articles to identify if the procedure for the isolation, purification and characterization of the compounds from natural products was present. We want to emphasize that this is the first version of BIOFACQUIM; future versions will have natural products from more years, more peer-reviewed journals and more institutions, to achieve a database representative of the biodiversity of Mexico.

With the module ’Wash’, from the molecular operating environment (MOE) program version 2018 [14], the database was curated. This was done to normalize and collect the most relevant information from the molecules. The data curation involved the elimination of salts, the adjustment of the protonation states, the optimization of the geometry by energy minimization and the elimination of the duplicated molecules. The default settings of the ‘Wash’ module were used.

### 2.2. Reference Data Sets

In order to characterize the diversity of BIOFACQUIM and to explore its coverage in chemical space, seven compound databases of broad interest in drug discovery were used as references. The structure files used in this work were taken from previous comparisons and chemoinformatic analyses of natural products [15]. The structures of the reference compounds were curated using the same procedure described to prepare BIOFACQUIM. Table 1 summarizes the reference databases and the number of compounds. Of note, the reference collections include seven data sets of natural products.

### 2.3. Molecular Properties of Pharmaceutical Relevance

The curated BIOFACQUIM database was characterized by calculating six physicochemical properties of therapeutic interest, namely: molecular weight (MW), octanol/water partition coefficient (SlogP), topological surface area (TPSA), number of rotatable bonds (RB), number of H-bond donor atoms (HBD) and number of H-bond acceptor atoms (HBA). The statistical analysis was done, with the program DataWarrior [16], by calculating the mean, median and standard deviation of the calculated properties. Based on these statistics BIOFACQUIM was further compared with other natural products databases (NuBBE_DB_, cyanobacteria, fungi, marine, and MEGx), approved drugs, and semisynthetic compounds (NATx) (Table 1).

### 2.4. Scaffold Content

Scaffold content analysis enabled us to identify the most frequent scaffolds in compound data sets and, in this work, to compare the scaffolds containing approved drugs with those containing natural products. The scaffold content analyses also enabled us to identify potential novel scaffolds. The most frequent core molecular scaffolds of BIOFACQUIM were computed using the definition described by Bemis and Murcko [17], in which the core scaffold is obtained by systematically removing the side chains of the compounds. The most frequent scaffolds in BIOFACQUIM were compared with data from the literature (vide infra).

### 2.5. Visual Representation of Chemical Space

In order to generate a visual representation of the chemical space of BIOFACQUIM, two visualization methods were used: principal component analysis (PCA) and *t*-distributed stochastic neighbor embedding (*t*-SNE). PCA reduces data dimensions by geometrically projecting them onto lower dimensions called principal components (PCs). The first PC is chosen to minimize the total distance between the data and its projection on the PC and to maximize the variance of the projected points.

*t*-SNE is a nonlinear dimension reduction in which Gaussian probability distributions over high-dimensional space are constructed and used to optimize a Student *t*-distribution in low-dimensional space. The low-dimensional space maintains the pairwise similarity to the high-dimensional space, leading to a clustering on the embedding space without any significant loss of structural information. Further details of each visualization method of the chemical space are discussed elsewhere [18,19]. In this work, for *t*-SNE, subsets of compounds were retrieved from large reference data sets (Table 1), namely: 40 % of the Marine, MEGx, and NuBBE_DB_ data sets (2501, 1641, and 886 compounds, respectively). For NATx and approved drugs, 1000 molecules were used. For cyanobacteria metabolites and fungi data sets the entire databases were employed (473 and 206 compounds, respectively).

### 2.6. Global Diversity: Consensus Diversity Analysis

Since the chemical diversity strongly depends on the structure representation, it is practical to consider multiple representations for a complete, global assessment. To this end, consensus diversity (CD) plots have been proposed as simple two-dimensional graphs that enable the comparison of the diversity of compound data sets using four sets of structural representations [20]; these are typically the molecular fingerprints, scaffolds, molecular properties, and number of compounds. CD plots have been used to compare the diversity of natural products and other compound data sets [21]. Briefly, in a typical CD plot the scaffold and fingerprint diversity are represented along the *y*- and *x*-axes, respectively. The diversity based on whole molecular properties of pharmaceutical interest is represented by a continuous color scale and the number of compounds is mapped into the plot using different size data points. Further details are provided elsewhere [20]. To generate the CD plot of this work, for the *y*-axis we used the area under the cyclic system recovery curve [22]. For the *x*-axis, we employed the median of the fingerprint-based diversity computed with MACCS keys (166-bits) and the Tanimoto coefficient. Both are established and are representative metrics of the scaffold and fingerprint-based diversity. Subsets of the compounds were retrieved from large reference data sets (Table 1), considering the size of the databases. For NATx, Marine, MEGx, NuBBE_DB_ and approved drugs, 2000, 1500, 1000, 800 and 700 molecules, respectively, were used. For cyanobacteria metabolites and fungi data sets, the entire databases were employed (473 and 206 compounds, respectively).

## 3. Results and Discussion

First, we present the results of the construction of the first proof-of-concept version of the BIOFACQUIM database followed by a first chemoinformatic characterization in terms of physicochemical properties, scaffold content, diversity and coverage in chemical space.

### 3.1. BIOFACQUIM Database

As described in the Materials and Methods section, after the first survey in Scopus with the researchers of the FQ, UNAM, three filters were applied to the eight selected journals. Each of the 92 scientific papers selected was analyzed individually to extract information about the natural products. Of note, in this manuscript we disclose the first version of BIOFACQUIM as a proof-of-concept collection in which current content may be biased by the type of compounds published by a research group (e.g., based on their expertise and/or the analytical techniques available to their groups) and the type of compounds and characteristics accepted for publication by a given journal (e.g., compounds with the biological activity of compounds with drug-like features). It is anticipated that these biases will be reduced as the content of BIOFACQUIM is updated in future releases, by increasing the number of research groups, number of journals and number of years covered (cf. the Conclusions section).

**Figure 1:**
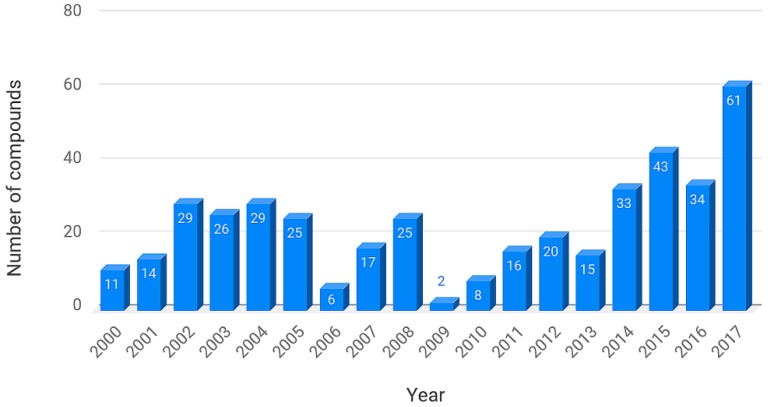
Distribution of compounds reported from 2000 to 2017, as contained in the first version of BIOFACQUIM. Compounds published in 2018 are not shown in this graph.

The current version of BIOFACQUIM contains the following information: identification number (ID), compound name, simplified molecular input line entry system (SMILES), reference (with the name of the journal, digital object identifier (DOI) number and publication year), kingdom (Plantae or Fungi), genus, species, and geographical location of the collection of the natural product. In addition, the biological activity, if it was reported in the publication, has been included. The current and first version of BIOFACQUIM has 423 compounds. It should be noted that 316 compounds were isolated from 49 different plant genera, 98 were isolated from 19 genera of fungi, and nine compounds were isolated from Mexican propolis (a sticky dark-colored hive product collected by bees from living plant sources). Figure 1 shows the distribution of compounds per year reported since the year 2000, as contained in the first version of the chemical data set. The compounds in the database that were published in 2018 are not included in Figure 1.

**Figure 2:**
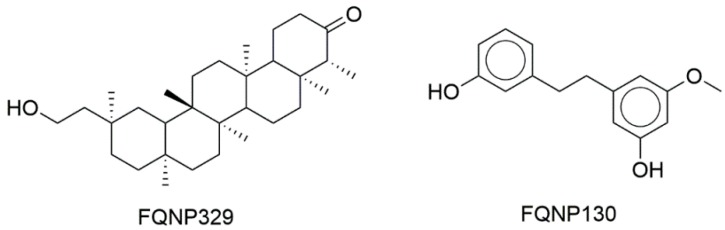
Select compounds contained in BIOFACQUIM.

Figure 2 shows the chemical structures of representative compounds from the first version of BIOFACQUIM (discussed further below).

### 3.2. Molecular Properties

**Figure 3:**
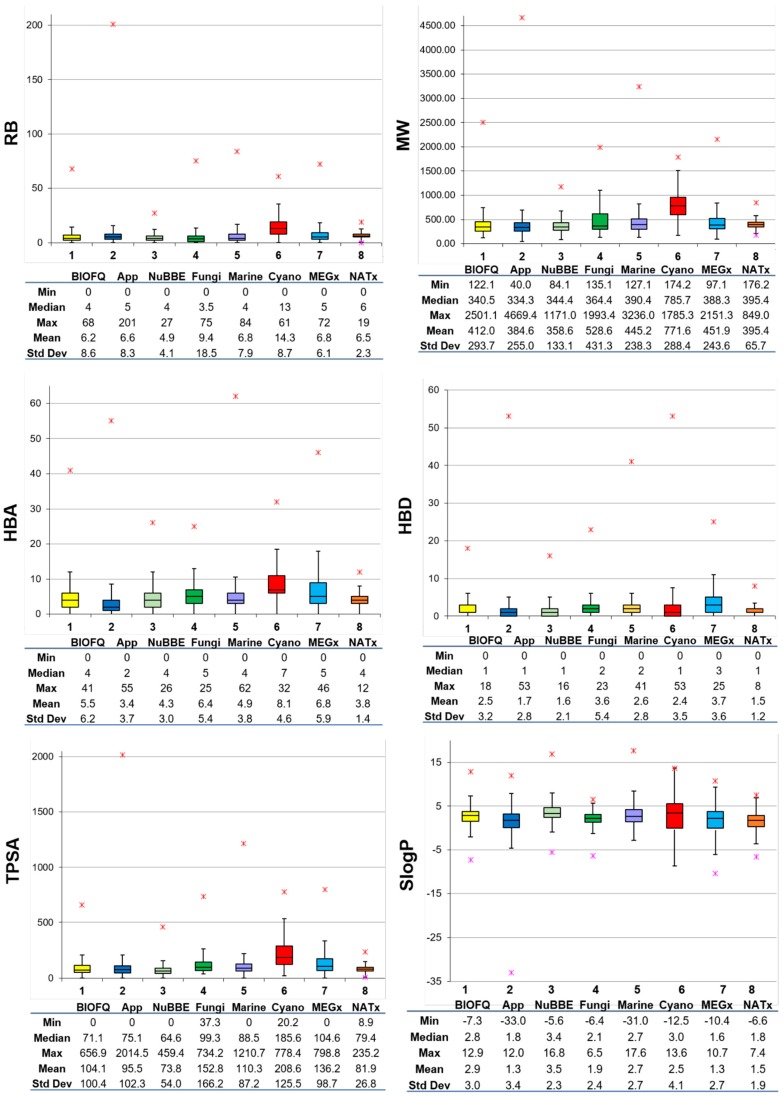
Box plots for the physicochemical properties of BIOFACQUIM (BIOFQ) and reference data sets (Table 1). The boxes enclose data points with values within the first and third quartile. The red asterisks indicate outliers. Summary statistics are included below each plot. RB: number of rotatable bonds; MW: molecular weight; HBA: number of H-bond acceptor atoms; HBD: number of H-bond donor atoms; TPSA: topological surface area; SlogP: octanol/water partition coefficient.

Figure 3 shows box plots of the distribution of the six calculated physicochemical properties (vide supra) calculated for BIOFACQUIM. For comparative purposes, the box plots also include the distribution of the same properties of the seven reference data sets that were retrieved from the literature [15]. The corresponding violin plots are shown in the Supplementary Figure S3. The three main molecular properties, size, flexibility, and molecular polarity, are described by MW, RB, and SlogP, TPSA, HBA, and HBD, respectively. In these plots, the boxes enclose the data points with values within the first and third quartile; the line that divides the box denotes the median of the distributions. The lines above and below indicate the upper and lower adjacent values. The red asterisks indicate the data points with values beyond the upper and lower adjacent values. Summary statistics are presented at the bottom of the box plots. The figure also includes a table below each box plot with the maximum, median, mean, standard deviation and minimum values for each property and each data set.

According to Figure 3 (and the violin plots in the Supplementary Material), based on the mean of RB, BIOFACQUIM compounds have comparable flexibility to approved drugs. The figure also shows that, except for cyanobacteria metabolites, all databases have a median of up to five rotatable bonds (including approved drugs). The median and mean MW of BIOFACQUIM are 340.5 and 412 g/mol, respectively. Notably, BIOFACQUIM and NuBBE_DB_ have the most similar MW profile compared to drugs. BIOFACQUIM has a median of 4 HBA, the same as that of the NuBBE_DB_ and Marine data sets. Furthermore, BIOFACQUIM has a very similar profile of HBA compared to MEGx. Comparing HBD, BIOFACQUIM, NuBBE_DB_, NATx, and cyanobacteria have the same median values, with similar profiles to approved drugs and higher standard deviations than approved drugs. Regarding TPSA, the compounds in BIOFACQUIM are those that share the closest values to the approved drugs. It should be noted that the cyanobacteria metabolite set has the largest distribution and the highest mean values of TPSA, being the double of the mean of the approved drugs. The distribution of the SlogP values indicates that, overall, natural products are slightly more hydrophobic than approved drugs.

Taking together the results of the general profile of the properties, it can be concluded that the current version of BIOFACQUIM is, in general, most similar to the NuBBE_DB_ and Fungi data sets. This outcome is in agreement with the findings that, while assembling BIOFACQUIM and analyzing the source papers in detail, the compounds were mostly isolated from plants and fungi.

### 3.3. Scaffold Content

**Figure 4:**
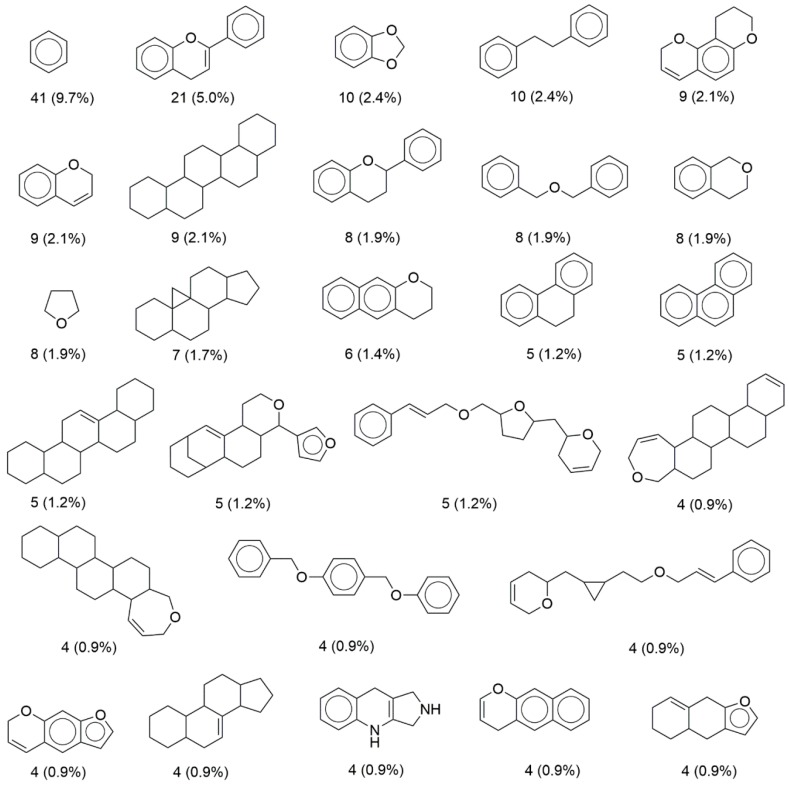
Most frequent scaffolds in BIOFACQUIM. The frequency and percentage are shown. The 27 scaffolds shown in the figure contain half of the total compounds in the database (50.6%).

Figure 4 shows the 27 most populated molecular scaffolds in BIOFACQUIM that included half (50.6 %) of the 423 compounds making up the database. Aside from benzene which is also frequent in several other compound databases [21], the second most frequent scaffold was a flavan-related scaffold (5 %), followed by 1,3-benzodioxole and dibenzyl core scaffolds (2.4 %). Interestingly, the last three frequent scaffolds in BIOFACQUIM are not the most frequent in other databases of natural products [15].

### 3.4. Chemical Space

As explained in the Materials and Methods section, a visual analysis of the chemical space of BIOFACQUIM was done with two visualization methods, PCA and *t*-SNE. The visual representation with PCA was based on the physicochemical properties while the visualization with *t*-SNE was based on the molecular topological fingerprints.

#### 3.4.1. Visual Representation Based on Properties

Using the program KNIME [23], we did a visual comparison of the chemical space of BIOFACQUIM and the reference databases. We used the “Normalizer” node in KNIME which gives a linear transformation of all values, the minimum and maximum of each database. Then, PCA was applied to reduce the dimensionality of the six calculated physicochemical properties and to compare BIOFACQUIM with the reference collections (vide supra, Table 1).

**Figure 5:**
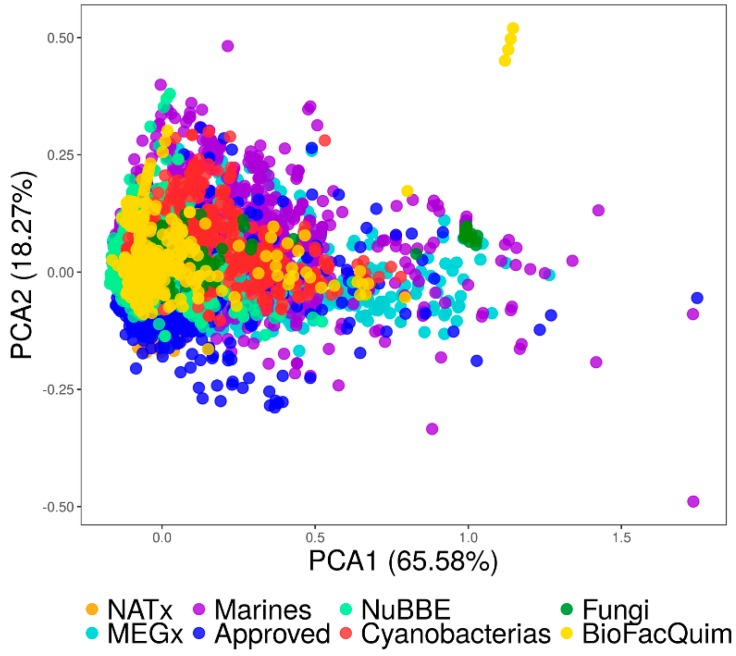
Visual representation of the chemical space based on the physicochemical properties of eight data sets. BIOFACQUIM (423 compounds, yellow); fungi metabolites (206 compounds, green); cyanobacteria metabolites (473 compounds, red); NuBBE_DB_ (2214 compounds, light green); NATx (26318 compounds, orange); MEGx (4103 compounds, blue); marine metabolites (6253 compounds, lilac); US Food and Drug Administration (FDA)-approved drugs (1806 compounds, dark blue).

Figure 5 shows a visual representation of the property-based chemical space. Table S1 in the Supplementary Material summarizes the corresponding loadings and eigenvalues for the first three PCs. The first two PCs capture 84% of the variance while the first three recover 92% of the variance. Table S1 shows that for the first PC, the larger loadings corresponded to SlogP, followed by RB, whereas for the second PC the largest loading corresponded to HBD.

The visual representation of the chemical space in Figure 5 indicates that some of the natural product compounds occupy the same space as the already approved drugs. It also shows that there are molecules in BIOFACQUIM and the Marine set that cover neglected regions of the currently drug-like chemical space. Finally, Figure 5 suggests that BIOFACQUIM shares the chemical space of almost all Fungi and NuBBE_DB_.

#### 3.4.2. Visual Representation Based on Molecular Fingerprints

**Figure 6:**
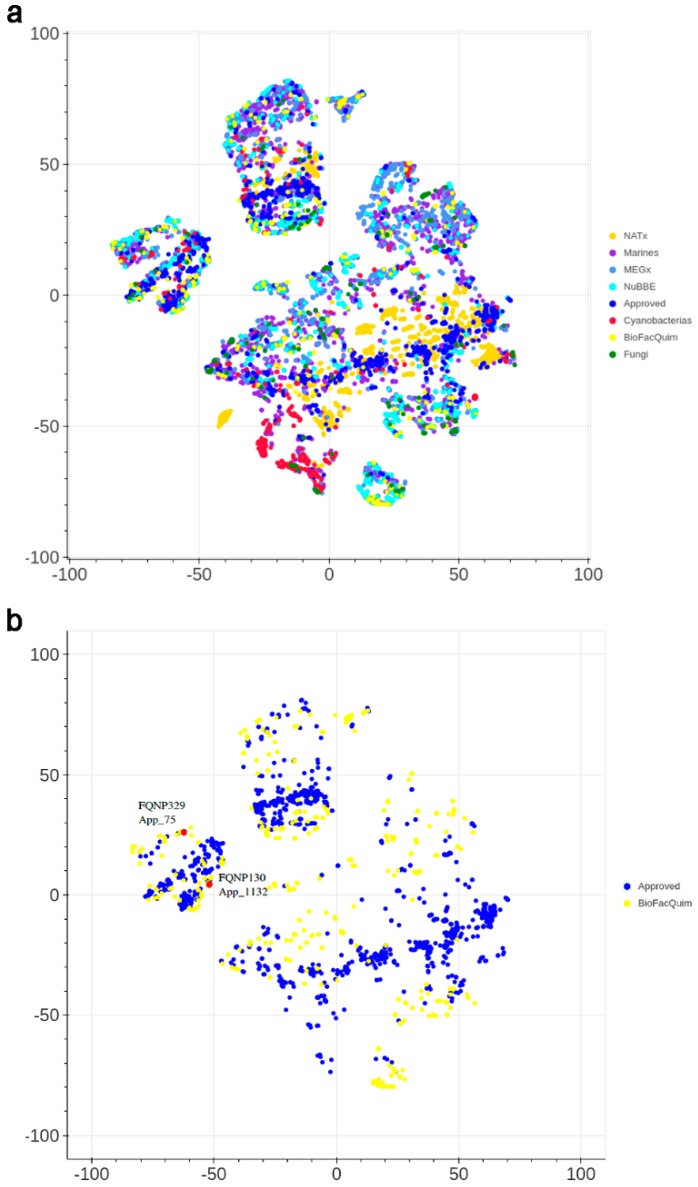
Visual representation of the chemical space of BIOFACQUIM compared with: (**a**) all reference data sets; and (**b**) approved drugs. The visualization was generated using *t*-distributed stochastic neighbor embedding (*t*-SNE) based on topological fingerprints. The red dots indicate the position of two representative compounds of BIOFACQUIM that are very similar to approved drugs.

Figure 6 shows a visual representation of the chemical space of the current version of BIOFACQUIM based on topological fingerprints using *t*-SNE (see Materials and Methods). Figure 6a compares BIOFACQUIM with all other reference data sets. Figure 6b shows a comparison of BIOFACQUIM with approved drugs. Figure 6a shows three main groups or clusters in which all the databases have compounds. The clusters indicate that the visualization method and the fingerprints can distinguish three major core structures that would have detailed variations in the structure. Figure 6b indicates that there are compounds in BIOFACQUIM with high structural similarity to approved drugs. Notable examples are the compounds FQNP329 (chemical structure in Figure 2), similar to ethinylestradiol (App_75), and FQNP130, similar to choline (App_878). Other comparisons with *t*-SNE are shown in Figure S3 in the Supplementary Material.

Based on the assessment of the chemical space, in particular the position of BIOFACQUIM relative to other reference libraries in chemical space, it can be concluded that the compounds in BIOFACQUIM are very similar to drugs, based on their physicochemical properties (PCA) and structural fingerprints (*t*-SNE). Therefore, the chemical space analysis further supports the use of BIOFACQUIM in drug discovery projects.

### 3.5. Global Diversity: Consensus Diversity Analysis

**Figure 7:**
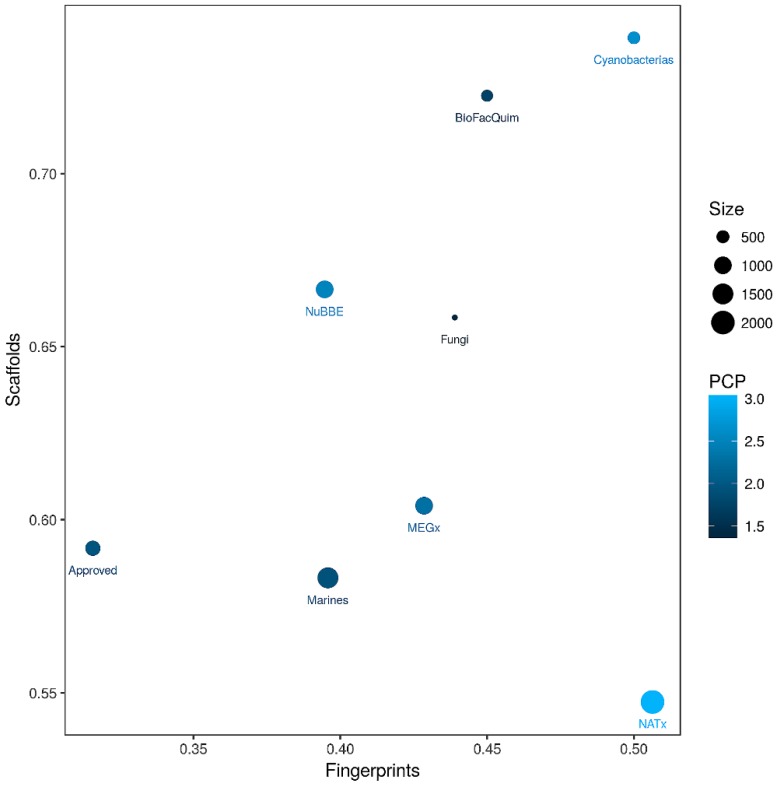
Consensus Diversity Plot comparing the global diversity of BIOFACQUIM with other natural product databases. The structural diversity (fingerprint diversity), calculated with the median Tanimoto coefficient of MACCS keys fingerprints, is plotted on the *x*-axis. The scaffold diversity of each database was defined as the area under the curve (AUC) of the respective scaffold recovery curves and is represented on the *y*-axis. The diversity, based on physicochemical properties (PCP), was calculated with the Euclidean distance of six scaled properties (SlogP, TPSA, MW, RB, HBD and HBA) and is shown on a color scale. The distance is represented with a continuous color scale from light blue (more diverse) to dark blue (less diverse). The relative size of the data set is represented with the size of the data point, smaller data points indicate compound data sets with fewer molecules.

As elaborated in the Materials and Methods section, a CD plot was used to compare the diversity of BIOFACQUIM with the diversity of the reference data sets, based on molecular fingerprints, scaffolds, and whole (physicochemical) properties. Figure 7 shows the CD plot, representing the MACCS keys/Tanimoto similarity on the *x*-axis. Here, lower values indicate larger fingerprint-based diversity (further details of the fingerprint-based diversity assessment are presented in Figure S1 in the Supplementary Material). The *y*-axis of the CD plot represents the scaffold diversity where lower values (the area under the scaffold recovery curve—see Table S2 in the Supplementary Material) indicate higher scaffold diversity. The property-based diversity of BIOFACQUIM and each database was calculated as the Euclidean distance of the scaled properties. The values are represented on the color CD plot with data points on a continuous color scale. The darker color represents lower diversity while lighter colors represent higher diversity. Finally, the relative size of the databases is represented with different point sizes, where smaller data points indicate data sets with less number of molecules. The CD plot in Figure 7 shows that BIOFACQUIM and Cyanobacteria are found in the area representing low diversity of both scaffold and fingerprints. This may be attributed to the fact that this is the first version of the database. Regarding the diversity, based on physicochemical properties, the cyanobacteria metabolites were observed to have more diversity (e.g., lighter blue data point in Figure 7) than BIOFACQUIM. This is consistent with the analysis of the box plots discussed in Section 3.2. Figure 7 also indicates that approved drugs have high scaffold and fingerprint diversity that is consistent with previous reports [20,21].

## 4. Conclusions

BIOFACQUIM is a compound database of natural products from Mexico being constructed, curated and maintained by an academic group. The first and current version of BIOFACQUIM includes 423 compounds reported over the past 10 years at the School of Chemistry of the National Autonomous University of Mexico (UNAM). The compound database contains the chemical name, SMILES notation, reference (with name of the journal, year of publication and DOI number), kingdom (Plantae or Fungi), genus and species of the natural product, and geographical location of the collection. In addition, the biological activity, if it was reported in the publication, was included. The chemoinformatic characterization and analysis of the coverage and diversity of BIOFACQUIM in chemical space suggest broad coverage, overlapping with regions in the drug-like chemical space. The analysis also indicated that there are compounds in BIOFACQUIM with chemical structures very similar to drugs approved for clinical use that could, based on the similarity principle, be of pharmaceutical interest. Similar to other natural product databases, BIOFACQUIM can be used, via virtual screening, to identify potential lead compounds or starting points for additional optimization. The database is freely accessible through the website BIOFACQUIM Explorer, version 1.0 (https://biofacquim.herokuapp.com) and is part of the initiative D-TOOLS, described in detail elsewhere [24]. Compounds in BIOFACQUIM are also available from ZINC15 at http://zinc15.docking.org/catalogs/biofacquimnp/

One of the major objectives of this work, currently in progress, is to augment the size of BIOFACQUIM by expanding the search to other universities and research centers in Mexico, increasing the number of years and the number of scientific international peer-reviewed journals covered (with DOI number available). A second major objective of this work is to continue improving and maintaining the web-based interface BIOFACQUIM Explorer following general guidelines for the development and maintenance of public biological databases [25].

## Figures and Tables

**Table 1:** Reference databases [15] compared for BIOFACQUIM.

Database	Size ^a^
Approved drugs	1806
Cyanobacteria metabolites	473
Fungi metabolites	206
Marine	6253
MEGx	4103
Semi-synthetics (NATx)	26,318
NuBBE_DB_	2214

^a^ Number unique compounds after data curation.

## Supplementary Materials

The following are available online at http://www.mdpi.com/2218-273X/9/1/31/s1. Table S1. Loadings for the first three principal components of the property space of eight databases. Table S2. Statistics of the cyclic system recovery curves for BIOFACQUIM and the reference data sets. Figure S1. Distribution of the pairwise similarity values calculated for BIOFACQUIM and the reference data sets computed with MACCS keys (166-bits) and the Tanimoto coefficient. Figure S2. Visual representation of the chemical space of BIOFACQUIM generated with *t*-SNE. Figure S3. Violin plots for the physicochemical properties of BIOFACQUIM and reference data sets.
