# Supplementary material for: BIOFACQUIM: A Mexican Compound Database of Natural Products
Source: Biomolecules. 2019 Jan 17;9(1):31. doi: 10.3390/biom9010031 (PMC6358837; doi:10.3390/biom9010031)
Supplement: Supplementary file 1 [file biomolecules-09-00031-s001.pdf]

SUPPLEMENTARY MATERIAL

BIOFACQUIM: A Mexican compound database of natural products

B. Angélica Pilón-Jiménez, Fernanda I. Saldívar-González, Bárbara I. Díaz-Eufracio, José L. Medina-Franco

Contents

		Page
**Table S1**	Loadings for the first three principal components of the property space of eight databases.	S2
**Figure S1**	Distribution of the pairwise similarity values calculated for BIOFACQUIM and the reference data sets computed with MACCS keys (166-bits) and the Tanimoto coefficient.	S3
**Table S2**	Statistics of the cyclic system recovery curves for BIOFACQUIM and the reference data sets.	S4
**Figure S2**	Visual representation of the chemical space of BIOFACQUIM compared with: **a**) Fungi, **b**) NATx, **c)** Cyanobacterias, **d)** MEGx, **e)** NuBBEDB, **f**) Marines; Figure generated with t-SNE.	S5
**Figure S3**	Violin plots for the physicochemical properties of BIOFACQUIM and reference data sets.	S6

Table S1. Loadings for the first three principal components of the property space of eight databases

Principal Component	PC1	PC2	PC3
Eigenvalue	1.98	1.05	0.71
Cumulative eigenvalue (%)	65.58	83.85	92.15
SlogP	0.18	-0.86	0.23
TPSA	-0.49	0.04	0.21
MW	-0.45	-0.31	0.13
HBA	-0.45	-0.04	0.47
HBD	-0.44	0.23	-0.08
RB	-0.37	-0.33	-0.81

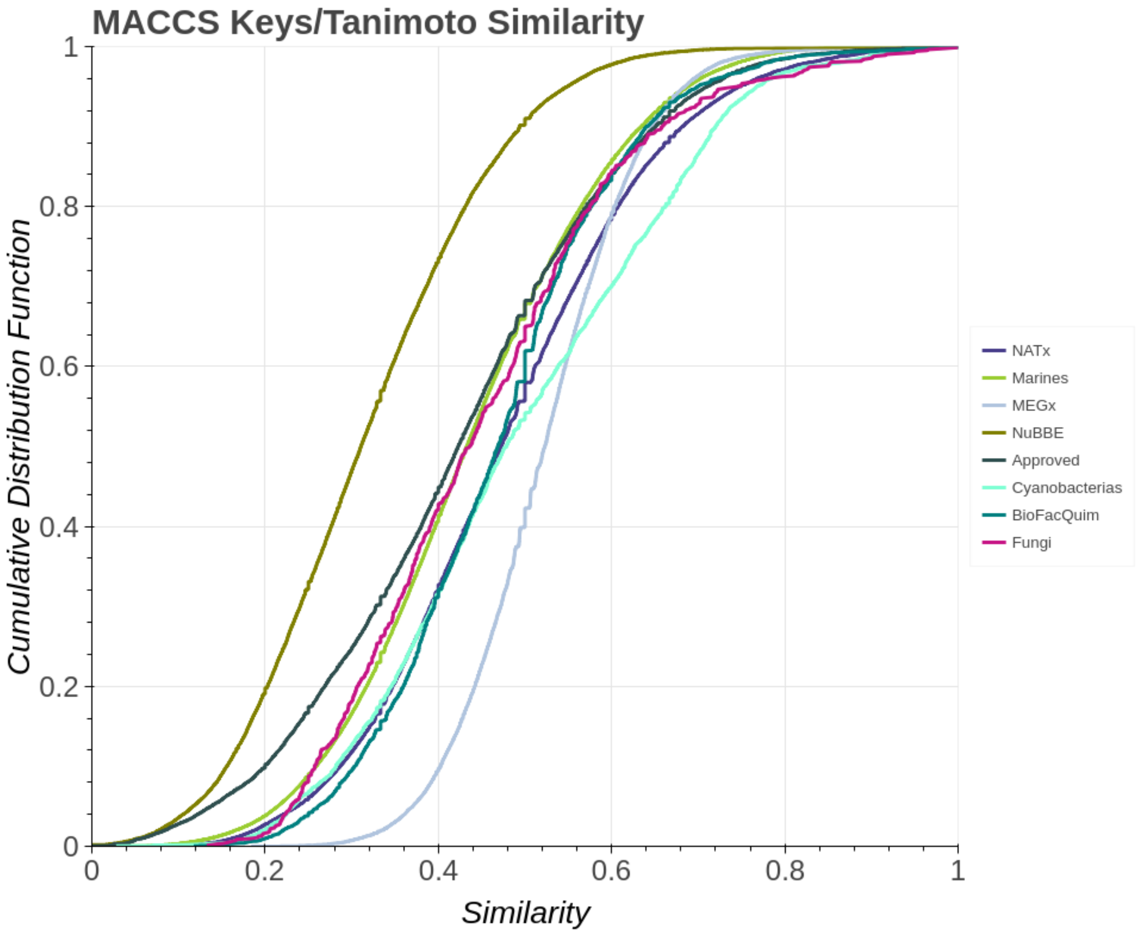

	NATx	Marines	MEGx	NuBBE	Approved	Cyanobacterias	BIOFACQUIM	Fungi
**MIN**	0.06	0.0	0.18	0.0	0.0	0.03	0.12	0.13
**1Q**	0.0	0.0	0.0	0.0	0.0	0.0	0.0	0.0
**MEDIAN**	0.0	0.0	1.0	0.0	0.0	0.0	0.0	0.0
**MEAN**	0.48	0.44	0.52	0.32	0.42	0.5	0.47	0.45
**3Q**	0.58	0.54	0.59	0.41	0.54	0.63	0.55	0.55
**MAX**	1.0	1.0	1	1.0	1.0	1.0	1.0	1.0
**STD**	0.15	0.14	0.1	0.13	0.17	0.17	0.13	0.16

Figure S1. Distribution of the pairwise similarity values calculated for BIOFACQUIM and the reference data sets computed with MACCS keys (166-bits) and the Tanimoto coefficient.

Table S2. Statistics of the cyclic system recovery curves for BIOFACQUIM and the reference data sets

DB	PCP<br>EDmedian	FP<br>Tmedian	Scaffold<br>AUC	Relative size
Approved	1.96	0.32	0.59	699
BIOFACQUIM	1.74	0.45	0.72	423
Cyanobacterias	2.64	0.50	0.74	473
Fungi	1.39	0.44	0.66	206
MEGx	2.28	0.43	0.60	1000
Marines	1.93	0.40	0.58	1500
NATx	3.04	0.51	0.55	2000
NuBBE	2.51	0.39	0.67	1000

Databases (DB), physicochemical diversity (PCP), Euclidean distance (ED), fingerprint (FP), Tanimoto coefficient (T), area under the curve (AUC).

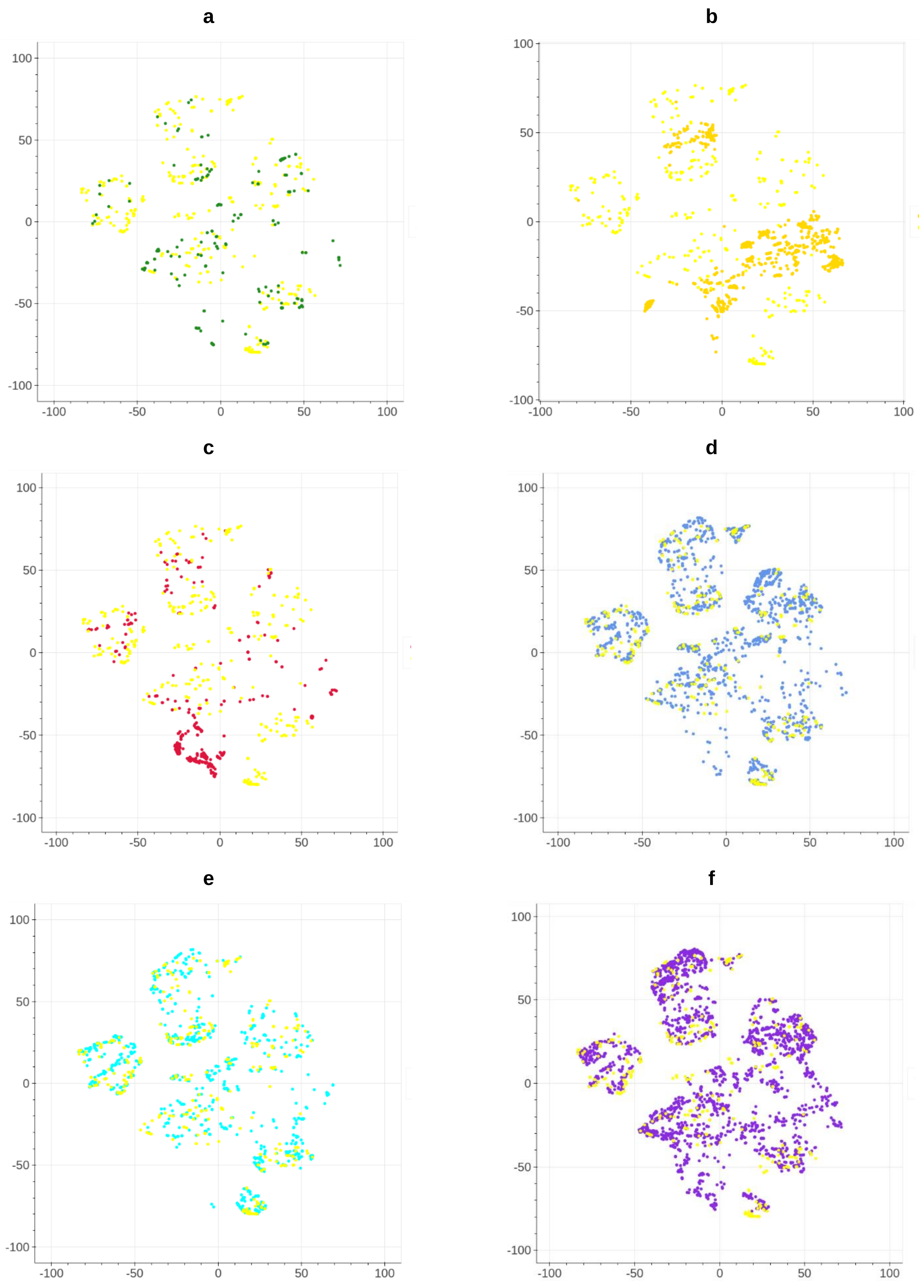

Figure S2. Visual representation of the chemical space of BIOFACQUIM compared with: **a**) Fungi, **b**) NATx, **c)** Cyanobacterias, **d)** MEGx, **e)** NuBBE_{DB}, **f**) Marines. Figure generated with t-SNE.

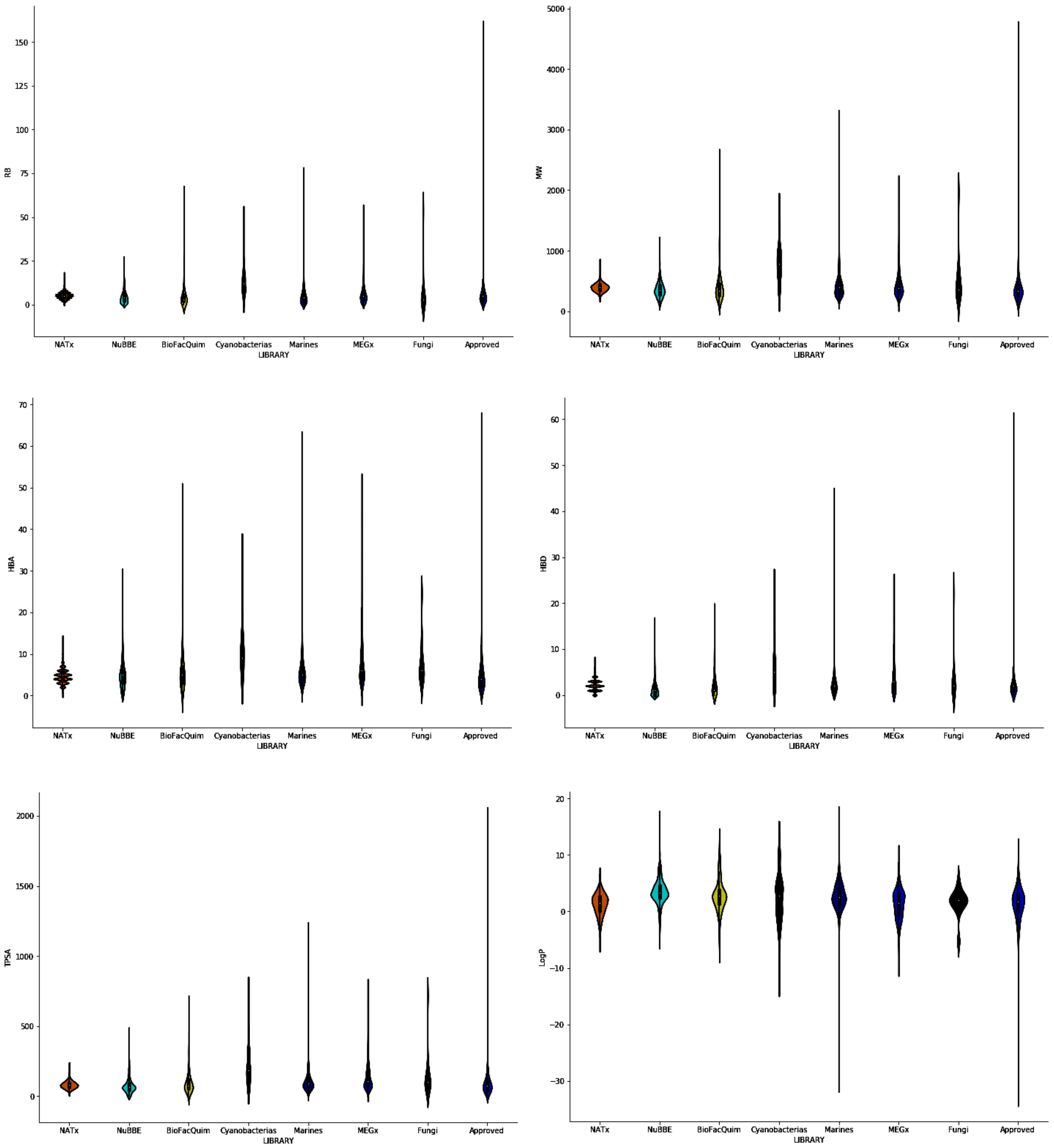

Figure S3. Violin plots for the physicochemical properties of BIOFACQUIM and reference data sets.
